# The Unprecedented Success of Chimeric Antigen Receptor T-Cell Therapy in the Treatment of Hematological Malignancies

**DOI:** 10.7759/cureus.59951

**Published:** 2024-05-09

**Authors:** Sargam Dhaliwal, Fatehpal S Gill, Pousette Hamid

**Affiliations:** 1 Internal Medicine, California Institute of Behavioral Neurosciences & Psychology, Fairfield, USA; 2 Neurology, California Institute of Behavioral Neurosciences & Psychology, Fairfield, USA

**Keywords:** car t-cell therapy, adoptive cell therapy, immunotherapy, hematological malignancies, chimeric antigen receptor

## Abstract

Chimeric antigen receptor (CAR) therapy is one of the most unprecedented advancements in the treatment of hematological malignancies, especially B-cell malignancies. The fundamental notion behind the success of this therapy is to generate a synthetic protein (CAR) capable of redirecting T lymphocytes to act against cancer cells. New insights into the genetic and molecular base of hematological malignancies have more recently given rise to the development of targeted treatments. CAR T-cell therapy is one of these immunological treatment techniques that has recently received a lot of attention and paved a light of hope for the effective cure of relapsed and refractory hematological malignancies and some solid malignancies. Researchers of today might not know what the future holds for CAR T-cell therapy, but from whatever research has been done so far, this therapy has proven to be a success despite its limitations, and it can be assumed that the spectrum of its application is expanding with each passing day.

## Introduction and background

"After immunotherapy, they didn't find any cancer at all" (Jimmy Carter, former president of the United States). Former US President Jimmy Carter reported in August 2015 that his melanoma progressed to his liver and brain. Carter's advanced melanoma would have been untreatable a few years ago. Carter's doctors, fortunately, utilized a relatively new strategy that combined radiation with immunotherapy, a form of treatment that increases the immune system's power to prevent, control, and treat malignancies [1]. Success stories like Jimmy Carter's are not rare after the advent of different forms of immunotherapy. Cellular immunotherapies have revolutionized oncology by harnessing and enhancing the immune system's natural ability to fight cancer.

In the 1890s, William Coley noted the remission of sarcoma after severe bacterial infections. He is generally credited with the first observations that immune system engagement had antitumor effects. Jim Alison and Tasuku Honjo were awarded the 2018 Nobel Prize in Physiology or Medicine in 2008 for their contributions to tumor immunity research. According to the study's findings, tumor immunotherapy has a great deal of potential for treating various types of tumors. Recently, a lot of interest has been seen in chimeric antigen receptor (CAR) T-cell (CAR-T) therapy as a type of tumor immunotherapy. Adoptive T-cell therapy (ACT), a form of immunotherapy, was developed as a result of the key discovery that hematopoietic stem cell transplantation employing syngeneic donors was less effective than sibling donors at preventing relapse of leukemia [2,3].

Hematological malignancies have made tremendous progress in treatment over the last few decades, but they still have a high morbidity and mortality rate. Chemotherapy, radiotherapy, and stem cell transplantation have traditionally been used to treat hematological malignancies. Although conventional treatments like chemotherapy and radiotherapy may provide short-term benefits, their invasiveness and biotoxicity can lead to unpleasant side effects. In addition, chemotherapy and radiotherapy are limited in their curative effects by multidrug resistance and multiple toxicities. Because of this, it is necessary to develop new and effective treatments for the condition.

**Figure 1:**
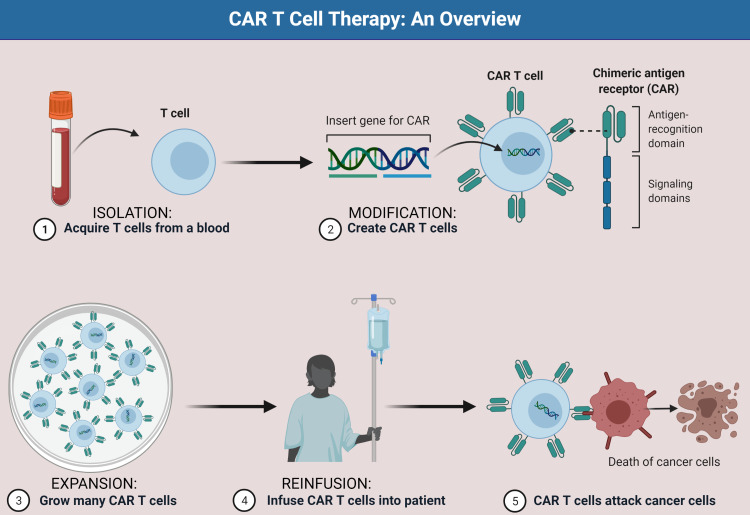
Step-by-step approach of CAR T-cell therapy Four major steps are involved in this process: First, the peripheral blood mononuclear cells are extracted from the patient's own blood. Second, modification is done by activating T cells and transducing CARs into activated T cells. Third, expansion of modified T cells is done in vitro to obtain a significant number of cells. Fourth, the modified and expanded T cells are re-infused in desired doses into the patient with a lower number of T cells. CAR: chimeric antigen receptor. This figure is an original illustration by the authors.

Immune-targeted therapy has emerged as a new treatment option for hematological malignancies in recent years, with more insights into the molecular genetic basis of these cancers. Immunotherapy development could be aided by a better understanding of the interaction between the patient's immune system and cancer cells. T cells have been shown to have a lot of potential for immunotherapy of hematological malignancies. CAR-T therapy, a form of ACT, is a reprogramming technique that involves extracting cells of the immune system from the affected persons or those willing to participate, after which gene editing and in vitro expansion are done, followed by re-injecting them back into patients (Figure 1). It has been widely used to treat T-cell senescence [4]. The effectiveness of ACT is improved when patients have enough effector cells with antitumor recognition capabilities [4].

ACT is one of the most recent advancements in immunotherapy. Adoptive transfer of genetically engineered T cells expressing chimeric antigen receptors is one promising ACT method. In 1989, Eshar proposed the first CAR theory, which proposed equipping T cells with CAR in order to direct them against a specific tumor antigen [5]. T-cell receptors that recognize predefined target Ag and introduce it into T-cells to activate their cytotoxicity against target cells are known as CARs. The tumor surface antigen is recognized by CAR-Ts in an antibody-like pattern that is independent of the major histocompatibility complex [6]. CAR-Ts can recognize a wide range of targets, including proteins, carbohydrates, and glycopeptides; as a result of this, these cells are able to kill tumor cells after recognizing the specific target antigen on them [7].

CD-19-targeted CAR-T therapies, which were approved by the US Food and Drug Administration (FDA) as a type of ACT, have shown great results for the treatment of hematological malignancies [8,9]. The FDA designated CTL019, a CD19-directed CAR-T treatment developed by the University of Pennsylvania, as a breakthrough therapy on July 1, 2014. Tisagenlecleucel was accepted for the treatment of children and young adults with relapsing or refractory acute lymphoblastic leukemia, and axicabtagene ciloleucel was approved for the treatment of non-Hodgkin's lymphomas by the FDA in 2017. This therapy has also been shown to be beneficial for other cancers, including non-small cell lung carcinoma, malignant pleural mesothelioma, metastatic renal cell carcinoma, and glioblastoma, but the effectiveness of the therapy is low in these cancers as compared to hematological malignancies.

The goal of this review is to gain an understanding of how CAR-T therapy can be used to treat various hematological malignancies, particularly refractory and relapsed cancers like acute lymphoblastic leukemia, acute myeloid leukemia, chronic myeloid leukemia, chronic lymphocytic leukemia, diffuse large B-cell lymphoma, Hodgkin's lymphoma, and multiple myeloma in both adult and pediatric patients and to discuss the prospects for CAR-T therapy in cancer treatment in the future.

## Review

CAR has a distinct structure. A CAR is composed of four domains: the extracellular domain, the hinge or spacer, the transmembrane (TM), and the intracellular domain. A particular antibody's single-chain variable fragment (ScFV) component, which is aimed against the target antigen, typically comprises the extracellular domain. The flexibility of the spacer/hinge domain, which is typically composed of IgG1, affects the extracellular domain and function of the CAR T cell. Primarily derived from CD8/CD28, the transmembrane domain affects CAR expression on the T cell membrane. The intracellular domain contains the CD3 signaling pathway, which, upon attaching to the target cell, activates the T cell. Co-stimulatory domains like CD28 and 4-1BB, which are employed to create the second and third generations of CAR T cells, can increase T cell proliferation, cytokine secretion, antitumor activity, and persistence by offering an additional signaling pathway [10].

**Figure 2:**
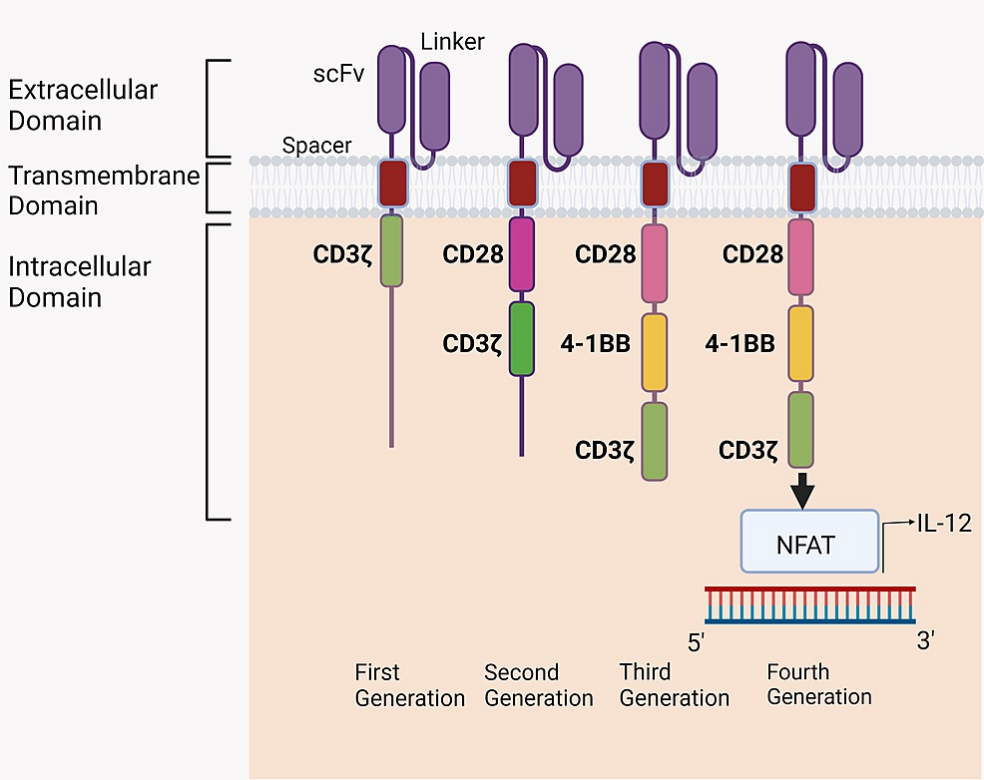
Showing different generations of CARs CAR: chimeric antigen receptor, NFAT: nuclear factor of activated T cells The figure is an original illustration by the authors.

There have been four generations of CAR T cells injected thus far (Figure 2). In the first generation (1G), the intracellular domain is the CD3 signaling chain, and the target recognition domain is the ScFV. The second generation (2G) has additional characteristics to the first generation, such as the presence of a co-stimulatory domain like 4-1BB (CD137) or CD28 as a secondary signal generator. It was possible to produce third-generation (3G) CAR T cells by integrating the co-stimulatory domains 4-1BB and CD28 [11]. Moreover, the fourth generation (4G), called T-cell redirected for universal cytokine killing (TRUCK T) or armored CAR T cell merges second-generation qualities (2G) with enhanced tumor efficiency, such as the production ability of cytokines. Several cytokines, including interleukin-15 (IL-15) and interleukin-12 (IL-12), have been used to boost CAR-T therapy against tumor cells.

Applications of CAR-T therapy in hematological malignancies 

*Acute Lymphoblastic Leukemia (ALL)* 

In ALL, malignant blasts in the bone marrow and undifferentiated cells proliferate excessively. Relapsed and refractory (r/r) B-ALL, in particular, can be treated with CAR T cells [12]. A transmembrane glycoprotein that regulates B cells in an antigen receptor-dependent manner, CD19 is expressed throughout the differentiation process of B cells but most prominently during the malignant transformation of B cells. Researchers are attracted to CD19 as a potential target for lymphoma treatment because of its unique characteristics. Two patients with r/r ALL were treated with anti-CD19 antibodies and CTL019 CAR T cells by Grupp et al. An examination of the bone marrow of the two patients revealed that the level of CTL019 CAR-T expansion was 1000 times higher than the transplantation level. In addition, CAR T cells were found to persist at high levels in the cerebrospinal fluid (CSF) for at least six months in both patients, and complete remission (CR) was observed in both patients [13]. In addition, Maude et al. used tisagenlecleucel (formerly CTL019) to treat r/r B cell ALL patients. Seventy-five patients received tisagenlecleucel injections, with an 81% complete remission rate (CRR) after three months. Flow cytometry was used to evaluate all of the patients who responded to the treatment, and the minimal residual disease (MRD) was found to be negative. Tisagenlecleucel was found to be present in the blood for up to 20 months [14].

In another study, 16 r/r B-ALL patients were treated with autologous T cells expressing the 19-28Z CAR, which is CD19 antigen-specific. After evaluating the results of IgH deep sequencing and using morphological criteria, the CRR was determined to be 88%. It is difficult to identify targets for ALL because leukemia cells express the same antigen as normal T cells. Cytokine release syndrome (CRS) is a common complication of CAR-T therapy for ALL [15].

Chronic Lymphocytic Leukemia (CLL)

The most prevalent adult leukemia is CLL. In patients suffering from multiple r/r CLL, the prognosis is poor. Targeting CD19 with CAR T cells is an effective way to treat CLL. It was developed by a team at the University of Pennsylvania that can bind to CD3zeta (receptor for T-cell antigen) and CD137 (a signal-transduction component) (a co-stimulatory receptor in T cells) [16]. It was found that after CLL patients received low-dose autologous CAR-Ts (approximately 1.5105 cells per kilogram of body weight), the number of CAR T cells increased by 1,000 times, and the tumor was totally cured. Moreover, for an additional six months, CAR was continuously expressed in the blood and bone marrow. Furthermore, this group presented the results of 14 patients with r/r CLL who received CAR-T therapy. They gave CTL019 T cells to patients with r/r CLL at a dose of 0.14 x 10^8^ to 11 x 10^8^ cells (median 1.6 x 10^8^ cells) and tracked the circulating CTL019 T cells for toxic effects, ability to respond, expansibility, and consistency. The total response rate of these treated CLL patients was 8/14 (57%) with four CRs and four partial remissions (PRs) [17]. The combination of CAR-T therapy with other therapies has shown some promise in treating CLL patients. Eight CLL patients were treated with CD19-targeted CAR T cells integrated with CD28 intracellular signaling domain (19-28 Z) after first-line chemotherapy with infliximab, according to Geyer et al. The objective response (OR) was observed in three out of eight patients, among whom clinical CR of two patients sustained more than 28 months. IL-6, IL-10, IL-2, and transformation growth factor (TGF) were increased in four patients after CAR T-cell infusion (CTI) showing signs of fever. It took 48 days for four patients to show evidence of CAR T cells after infusion [18]. According to a study by Gauthier et al., ibrutinib was effective against r/r CLL in 83% of patients after four weeks, and 61% of patients achieved MRD by IgH sequencing negative bone marrow response. The one-year progression-free survival (PFS) after CAR-T therapy was 38% and 50%, respectively, in cases treated with or without ibrutinib. It was well tolerated when ibrutinib and CAR T cells were used together [19]. Such studies show that CAR T cells are effective in treating chronic lymphocytic leukemia.

Non-Hodgkin's Lymphoma (NHL)

In recent years, despite significant advances in the treatment of NHL with chemotherapy, radiotherapy, and hematopoietic stem cell transplantation, the mortality rate has remained unchanged. New treatment approaches are essential for patients who are resistant to standard treatment regimens. Due to its remarkable success in treating r/r lymphoma, CAR-T therapy has recently attracted a lot of attention.

As the most common subtype of NHL, diffuse large B-cell lymphoma (DLBCL) has an aggressive clinical course. Two patients with relapsed DLBCL after autologous hematopoietic stem cell transplant were treated with CAR T cells expressing anti-CD20, but no clinical responses or obvious toxicities were observed [20]. A study by Kochenderfer et al. reported that anti-CD19 CAR T cells were used to treat 15 patients with advanced B-cell malignancies, and CR was achieved in four of seven chemotherapy-resistant DLBCL patients. In addition, 636 patients with r/r DLBCL were retrospectively analyzed in the SCHOLAR-1 study for efficacy and survival. Twenty-six percent (26%), 7%, and 6.3 months, respectively, were the overall response rate (ORR), complete response rate (CRR), and overall survival (OS) for the r/r groups [21]. CTL019 CAR-T therapy was also used in the treatment of 28 lymphoma patients in adults by Schuster et al. [22]. The CAR T cells used by Stirrups and colleagues to treat patients with refractory large B cell lymphoma were anti-CD19 [23]. To test their CAR T cells, they selected 111 patients with histologically proven large B-cell lymphoma, including DLBCL and primary mediastinal B-cell lymphoma, and infused them with anti-CD19 CAR T cells at 2 × 106 cells/kg body weight. According to an analysis of 101 patients with the intention to treat, 83 had OR, while 55% had CRs, while 28% had PRs.

Seven percent of all NHL cases are caused by mantle cell lymphoma (MCL). As a result of CAR-T therapy, MCL is curable. Till et al. performed a preclinical trial in four patients with relapse-indolent B cells and MCL using CD20-specific CAR T cells with co-stimulated domains CD28 and 4-1BB. As a result, this therapy was well tolerated, despite the fact that one patient experienced transient infusion symptoms. An objective PR occurred in one patient, and the disease relapsed 12 months after injection, and two patients showed no progress at 12 and 24 months [24]. Other subtypes of NHL have also been treated with CAR T cells by some scientists. They found that after treating them for two years with two or more treatment lines, 14 follicular lymphoma (FL) patients had progressed after receiving CTL019 in a phase IIa study [21]. Patients with refractory and aggressive B-cell NHL received KTE-C19, a single-chain antibody called FMC-63 that recognizes CD19 on tumor cells' surfaces in a study by Neelapu et al. Patients who completed treatment had an overall effectiveness rating of 79% and an overall efficacy rating (EFR) rating of 52% [25].

Hodgkin's Lymphoma (HL)

It arises from B cells and overexpresses CD30, which could be used as a target for therapy. One patient who was treated with anti-CD30 CAR T cells for more than two and a half years and another who was treated for almost two years achieved complete remission, according to a phase I study. Three other patients experienced transient stable disease (SD). A noteworthy finding in this study was the absence of toxicity from anti-CD30 CAR-T therapy [26]. On top of that, Wang et al. administered 1.5 107 CAR T cells per kilogram of weight to 11 patients with HL. Nine cases (82%) responded to treatment, one (9%) maintained continuous CR, one (46%) achieved PR, and three cases (27%) remained stable, according to the report. There was a tolerable infusion-related fever syndrome in each patient. After two weeks of infusion, one patient (9%) experienced self-limited arthralgia, myalgia, and swelling of both knees for five days [27]. It is clear from these results that CD30 has great potential in treating high-level HL.

Multiple Myeloma (MM)

In MM, monoclonal plasma cells proliferate in the bone marrow, causing an overabundance of monoclonal paraprotein (M protein), bone destruction, and displacement of other hematopoietic cell lines. CAR T cells that are anti-CD19 have a weak killing effect on MM cells, which can cause damage to healthy tissues [28]. It is, therefore possible, to treat MM by searching for specific targets expressed only on MM cells. According to Hosen and co-workers, the active conformation of integrin is a specific target for the treatment of MM. MMG49 was identified as the MM monoclonal antibody that specifically recognizes the integrin-7 molecule after screening more than 10,000 anti-MM monoclonal antibodies [29]. In this way, it was shown that the transduced cells from MMG49 did not harm normal hematopoietic cells but were effective against myeloma cells. BCMA, CD269, which is a B-cell maturation antigen, is another molecule that can be used to treat MM. A study by Xu et al. on a female patient who had been diagnosed with r/r MM and had received anti-BCMA CAR T cells showed to have experienced CR. Patients having a 7.6-month, disease-free survival rate, on the other hand, developed grade one CRS, which was characterized by fever and nausea and eventually led to the recurrence of MM [30].

Both normal and malignant plasma cells express high levels of CD138 (also known as syndecan-1), which has been categorized as an important target for many years. When transduced with CD138-specific chimeric antigen receptors, T cells from healthy donors and patients with MM were able to kill myeloma cell lines and primary myeloma cells in vitro and in vivo as reported by Sun et al. There was no off-target tumor cytotoxicity detected in normal epithelial or endothelial cells in preclinical studies [31]. CAR T cells exhibited rapid lysis of MM cell lines and primary multiple myeloma cells in vitro when they were treated with the anti-SLAMF7 antibody, huLuc63 (elotuzumab) [32]. CAR-T therapy for multiple myeloma has been considered a successful treatment option.

Acute Myeloid Leukemia (AML)

A form of hematological malignancy characterized by the presence of an excess of immature white blood cells in blood and bone marrow. Due to high relapse rate in both adult and pediatric AML, CAR-T therapy has shown positive results in some studies. AML can be treated with anti-CD123 CAR-T therapy [33]. A preliminary in vitro study found that anti-CD123 CAR-expressing killer T cells activated by cytokine exhibited excellent antitumor activity [32]. The CD33/Siglec-3 antigen has also been used to prepare allogeneic CAR T cells [33]. Trials with anti-CD33 CAR T cells on mice showed significant reductions in AML [34].

FLT3 has also been identified as an additional novel target for CAR-T therapy in AML [35]. Researchers found synergistic anti-leukemia effects between the FLT3 inhibitor crenolanib and FLT3 CAR T cells [35]. Additional evidence for leukocyte immunoglobulin-like receptor subfamily B member 4 (LILRB4)'s presence on the surface of AML was provided by John et al. [36]. They were able to successfully construct anti-LILRB4 CAR T cells that recognized AML cells [36]. CAR-T therapy targets for AML have not been tested in clinical trials, but they offer prospects for the development of AML therapies in the future.

Side effects and toxicities of CAR-T therapy

However, even though CAR-T therapy has yielded excellent results, it can be accompanied by some adverse side effects. Some toxicities associated with CTI may even be fatal. These side effects and toxicities have been discussed as follows:

Neurotoxicity

Although neurotoxicity has been observed in some clinical trials, the actual reason could not be established. Symptoms of CAR-T neurotoxicity include aphasia, confusion, delirium, difficulty finding words, myoclonus, and seizures, among others [37]. Six hours and three days after receiving autologous CAR T cells, Hu et al. reported neurological symptoms in a female with refractory/relapsed ALL. The patient was treated with methylprednisone until day 14, which resulted in a complete disappearance of the patient's symptoms [37]. Neurotoxicity is currently not managed with standard clinical interventions, but systemic corticosteroids may be used in severe cases. Dexamethasone may also be used due to its high central nervous system penetration [38].

CRS

Infusions of engineered T cells are associated with the most common toxicity CRS. Because of the intense activation of T cells, other immune cells become activated as well, creating a cytokine storm [39]. In addition to symptoms, such as fever, myalgia, vascular leak, and hypotension, CRS can also lead to multiple organ failures, eventually resulting in death. CRS is predisposed by a high tumor burden and a high dose of CAR T cells infused. The risk is also increased by strong T-cell proliferation and activation. High C-reactive peptide levels (over 20), IL-6, and interferon have been introduced as predictive biomarkers for CRS in recent years [40].

Anaphylaxis

As a result of the immunogenicity caused by ScFV derived from murine antibodies, anaphylaxis is immediate toxicity. Humanizing CAR protein components may reduce the risk of anaphylaxis. Since anaphylaxis is fatal, patients must be treated immediately [40].

Tumor Lysis Syndrome

Increased lactate dehydrogenase, uric acid levels, and potassium levels are associated with tumor lysis syndrome. Acute kidney injury can also occur as a result of tumor lysis syndrome. Hydration of the patient via intravenous route and Rasburicase treatment may help to lessen tumor lysis syndrome severity [41].

On-Target Off-Tumor Toxicity

It takes place when T cells are not able to distinguish between normal and tumor cells. Tumor-associated antigens (TAAs), which are expressed on both tumor and normal cells, account for the majority of CAR-T antigens. On-target off-tumor toxicity occurs when normal cells that express TAA are attacked [41]. B-cell aplasia is the most common form of on-target off-tumor toxicity. When using CD19 CAR-T therapies, this phenomenon is common. Since cardiac and pulmonary epithelial cells express the human epidermal growth factor receptor (HER) antigen, human epidermal growth factor receptor 2 (HER2)-targeted CAR T cells may cause cardiopulmonary toxicity [42]. The patient died because of lung damage after ERBB2 CAR-T therapy for colorectal cancer, according to a 2010 case report [43]. Tumor-specific antigens (TSAs), present only expressed on tumor cells, are preferred for CAR-T design in order to decrease the risk of on-target off-tumor toxicity.

Infections Associated With CAR-T Therapy

During CAR-T therapy, infections associated with CTI are relatively commonplace. Forty-two percent (42%) of 53 patients in a clinical trial using CD19 CAR T cells to treat relapsed B-cell ALL became infected within the first 30 days after CTI. Bloodstream infections (BSIs) were the most common type of infection [44]. Thirty-two patients in CR who survived the CTI developed infections between the 31st and 180th days. These infections were primarily viral, with respiratory viruses being the most prevalent. At the moment, the cause of CTI-related infections is unclear, and there is no unified treatment plan for the prevention and treatment of CTI-related infections. Some measures to prevent CTI-related infections include (a) paying attention to protection and avoiding cross-infection, (b) administering antibiotics and immunoglobulins to prevent and treat infections [45], (c) reducing the duration of glucocorticoid administration, (d) active treatment of cytokine release syndrome, and (e) reducing the input dose of CAR T cells. It is noteworthy that patients who receive CD19 CAR T cells are much less likely to develop life-threatening infections [44,45].

CAR-T therapy for solid tumors

An increased interest in treating solid tumors with CAR-T therapy stemmed from the phenomenal success of this therapy in treating blood cancers. However, solid malignancies posed some challenges to this therapy's application. This is due to factors such as the absence of adequate targets including CD19, as most target antigens are also expressed in normal tissues. In addition, solid tumors have a hostile immune-suppressive microenvironment that affects T cells, making them ineligible and ineffective targets, such as CD-19, and, finally, solid tumors are heterogeneous.

Although solid tumor CAR-T research is not as conclusive as hematological malignancy research, some studies have yielded promising results in tumors, such as non-small cell lung carcinoma, digestive tract tumors such as cholangiocarcinoma, epithelial ovarian carcinoma, glioblastoma, and various sarcomas. However, more scientific research is required to determine whether or not CAR-T therapy is as effective in treating these types of malignancies as hematological cancers.

Future prospectives 

Some of the ways and future directions by which the scope of CAR-T therapy can be broadened include the following:

Choice of Cell Types

It is preferable to use specific subtypes of less differentiated T cells, like T cells, to increase the number, longevity, and expansion time of CAR T cells in patients [46]. Moreover, it is believed that selecting CD4+/CD8+ T cells or central memory T cells as the starting cell population will lessen the variability of the final result [47,48]. As a part of their study, Blaeschke et al. administered memory T cells from the central nervous system and from stem cells to engineer CAR T cells that recognize CD19+ ALL. This study found that designing CAR T cells with a robust memory composition of T cells can increase the expansion of these cells by up to 100-fold. These CAR T cells were also more effective [49].

Combining Treatments Together

The combination of CAR-T therapy with immune checkpoint inhibition has recently been shown to improve its antitumor effect [50]. CAR-T therapy combined with chemotherapy and hematopoietic stem cell transplant (HSCT) may also improve patient outcomes and prolong patients' lives [51]. Combined with CAR-T therapy, these new treatment strategies could lead to enhanced antitumor efficacy.

Finding New Targets

As a part of the selection process for CAR T cells, tumor-specific antigens must be considered. New targets for hematological and solid malignancies have been identified, and improved antitumor efficacy and better results are expected.

Producing New Generations of CAR T cells

To increase the therapeutic use and efficacy of CAR T cells, additional changes and advancements are being developed despite the fact that they have been created in four generations thus far. Using clustered regularly interspaced short palindromic repeats (CRISPR/CAS9) technology, one of them is creating CAR T cells. A novel gene modification technology system called clustered regularly interspaced short palindromic repeats, or cas9, holds great potential for biological genome editing. Employing CRISPR/Cas9 for genome editing may result in more potent medicinal substances [52]. CRISPR/Cas9 technology has recently been used in conjunction with cancer immunotherapy to create the next generation of CAR T cells.

## Conclusions

From whatever research has been done so far, it can be concluded that CAR-T therapy has been found to be successful in the treatment of diseases, such as ALL, CLL, MM, Hodgkin's, and NHL. However, the greatest challenges that still limit the use of CAR-T therapy include its side effects, such as CRS, risk of relapse, neurotoxicity, and on-tumor off-target cytotoxicity. Researchers have constantly been looking for ways to overcome these side effects, and, as a result, most cases of CRS can now be controlled with an anti-interleukin-6 antibody, such as tocilizumab. Improving CAR-T production techniques, target selection, application of the latest technologies like CRISPR/Cas9 to this therapy, and clinical considerations can lead to the development of a number of genetically modified treatments for various malignancies and conditions. More extensive research is needed to overcome all the obstacles and expand the use of CAR-T therapy so that it can emerge as a new hope for patients with a variety of cancers, including solid tumors.
